# Adverse events associated with bimekizumab for moderate-to-severe plaque psoriasis: A retrospective, multicenter, post-hoc analysis

**DOI:** 10.1016/j.jdin.2025.02.001

**Published:** 2025-03-18

**Authors:** Nikki Zangenah, Maria Vazquez-Machado, Nicole Loranger, Soha Elshaboury, Kimberly Hashemi, Avery H. LaChance

**Affiliations:** a Department of Dermatology, Brigham and Women's Hospital, Boston, Massachusetts; b University of Massachusetts T. H. Chan Medical School, Worcester, Massachusetts; c Department of Specialty Pharmacy, Mass General Brigham, Burlington, Massachusetts; d Department of Dermatology, Medical University of South Carolina, Charleston, South Carolina

**Keywords:** adverse events, bimekizumab, biologics, epidemiology, IL-17, plaque psoriasis, post-hoc analysis, psoriasis

Bimekizumab, a monoclonal antibody selectively inhibiting interleukin (IL) 17A and IL-17F, was Food and Drug Administration–approved in 2023 for the treatment of moderate-to-severe plaque psoriasis in adults. Clinical trials have demonstrated a comparable safety profile to other IL-17A antagonists, with a slightly increased risk of fungal infection and the additional warning for suicidal ideation and behavior.^1^, ^2^, ^3^ Post-hoc analyses of adverse events (AEs) associated with bimekizumab outside of clinical trials are scarce, with no studies to date published in the United States.^4^^,^^5^ Our study aims to address this gap by characterizing the tolerability of bimekizumab among US adult psoriasis patients.

We conducted a retrospective cohort study of adult patients with psoriasis treated with bimekizumab at 2 academic centers from November 2023 to October 2024. Specialty pharmacy records were used to identify patients. Charts were reviewed in the electronic medical record to collect data on patient demographics, AEs, and treatment response. All patients were included in the analysis of tolerability, while efficacy data were collected only for patients on bimekizumab for 3 or more months. Descriptive statistics were calculated.

Fifty one and 38 patients met inclusion criteria for analysis of AEs and efficacy, respectively. Patient demographics included 33 patients (65%) with concomitant psoriatic arthritis and 4 (8%) with comorbid hidradenitis suppurativa (Table I). Overall, 71% (27/38) experienced improvement of psoriasis on bimekizumab, and 76% (19/25) had improvement in psoriatic arthritis (Table II). Key findings include 27 patients (53%) experiencing at least 1 AE related to bimekizumab. The most common AE was fungal infection (27%)—including thrush (18%), intertrigo (10%), and vulvovaginal candidiasis (6%)—with some patients developing multiple infections. Upper respiratory infection occurred in 10% of patients and COVID-19 in 6%. Other infections (8%) included pyelonephritis (4%), cellulitis (4%), and folliculitis (2%). Additional AEs included injection site reactions (10%), eczematous eruption (8%), worsening psoriasis (8%), and transaminitis (2%). One patient (2%) developed new-onset depression due to bimekizumab, but none reported suicidal ideation or behavior. Fifteen patients (29%) ultimately discontinued bimekizumab—7 (14%) for intolerable AEs, 7 (14%) for inadequate effect, and 2 (4%) for insurance reasons.

**Table I:** Demographic data

	Entire cohort (*n* = 51)
Age, mean (range)	55 (22-82) y
Sex, *n* (%)	
Female	25 (49)
Male	26 (51)
Race, *n* (%)	
Asian	3 (6)
Black or African American	2 (4)
White	43 (84)
Unknown	3 (6)
Ethnicity, *n* (%)	
Hispanic or Latino	4 (8)
Not Hispanic or Latino	44 (86)
Unknown	3 (6)
Insurance type, *n* (%)	
Commercial	40 (78)
Medicaid	1 (2)
Medicare	10 (20)
Diagnoses, *n* (%)	
Psoriasis	51 (100)
Psoriatic arthritis	33 (65)
Hidradenitis suppurativa	4 (8)

**Table II:** Bimekizumab treatment and adverse event data

	Entire cohort (*n* = 51)
No. of doses received, mean (SD)∗	5.0 (1.8)
Duration of treatment, mean (SD)	4.7 (2.7) mo
Improvement in psoriasis, *n* (%)	27 of 38 (71)
Improvement in psoriatic arthritis (if applicable), *n* (%)	19 of 25 (76)
Adverse events, *n* (%)	
Any adverse event(s)	27 (53)
Superficial fungal infection	14 (27)
Thrush	9 (18)
Intertrigo	5 (10)
Vulvovaginal candidiasis	3 (6)
Upper respiratory infection	5 (10)
COVID-19 infection	3 (6)
Other infection	4 (8)
Dental infection	1 (2)
Urinary tract infection	2 (4)
Skin or soft tissue infection	2 (4)
Depression or worsening mood	1 (2)
Suicidal ideation or behavior	0 (0)
Injection site reaction	5 (10)
Pain	4 (8)
Swelling	1 (2)
Redness	1 (2)
Headache	2 (4)
Fatigue	1 (2)
Nausea or vomiting	0 (0)
Hypersensitivity	0 (0)
IBD development or flare	0 (0)
Transaminitis	1 (2)
Eczematous eruption	4 (8)
Worsening psoriasis	4 (8)
Discontinued bimekizumab, *n* (%)	15 (29)
Adverse events	7 (14)
Inadequate effect	7 (14)
Insurance reasons	2 (4)

*IBD*, Inflammatory bowel disease; *SD*, standard deviation.

∗ All but 1 patient received weight-appropriate psoriasis dosing of bimekizumab; 1 patient received psoriatic arthritis dosing.

This study represents the largest observational post-hoc analysis of AEs associated with bimekizumab in adult psoriasis patients in the United States. Our results align with existing literature demonstrating fungal and upper respiratory infections as the most common AEs,^1^, ^2^, ^3^ reinforcing the value of directed counseling on these side effects when prescribing bimekizumab. The rate of thrush in our cohort was similar to clinical trials (12% to 19%), while upper respiratory infection was less common than the 24% to 39% seen in trials.^1^^,^^2^ The rate of serious AEs—including psychiatric symptoms, hepatic dysfunction, or development of inflammatory bowel disease—was exceedingly rare. Despite this, 14% of our cohort discontinued bimekizumab due to AEs, compared to 4% in clinical trials.^1^^,^^2^ This finding underscores that even milder side effects may limit patient acceptance and tolerability in real-life settings. Study limitations include variable documentation, infrequent follow-up, and recent bimekizumab initiation for some patients. Further investigations are warranted to continue evaluating real-world safety data for this novel therapeutic.

## Conflicts of interest

Dr LaChance has received research funding from Pfizer and Merck and consulting funds from J&J, MedaCorp, Guidepoint, and TD Cowen unrelated to the current manuscript. Authors Zangenah, Vazquez-Machado, Loranger, Elshaboury, and Dr Hashemi have no conflicts of interest to declare.
